# Efficacy and safety of dalpiciclib combined with endocrine therapy in elderly patients with HR^+^/HER2^-^ advanced breast cancer: a real-world study

**DOI:** 10.3389/fonc.2026.1789316

**Published:** 2026-08-18

**Authors:** Yan Li, Bingyu Li, Xiaoping Ma, Dan Liu, Li Li, Zhenhui Zhao, Xiner Zhang, Bing Zhao

**Affiliations:** Department of Breast Medicine, The Affiliated Tumor Hospital, Xinjiang Medical University, Urumqi, China

**Keywords:** dalpiciclib, efficacy, elderly, HR+/HER2- advanced breast cancer, safety

## Abstract

**Background:**

Dalpiciclib shows satisfactory efficacy and safety in patients with hormone receptor-positive (HR^+^)/human epidermal growth factor receptor 2-negative (HER2^-^) advanced breast cancer. Nevertheless, relevant real-world evidence is inadequate, particularly in elderly patients. This real-world study intended to explore the efficacy and safety of dalpiciclib plus endocrine therapy in elderly patients with HR^+^/HER2^-^ advanced breast cancer.

**Methods:**

This single-center retrospective study analyzed the data of 70 elderly patients (≥65 years) with HR^+^/HER2^-^ advanced breast cancer receiving dalpiciclib combined with endocrine therapy. The median (range) follow-up duration was 13.5 (1.7-29.4) months.

**Results:**

The objective response rate (ORR) was 14.3%, and the disease control rate was 94.3%. The ORR was higher in patients with first-line dalpiciclib-based therapy than in those with second-line therapy or above (24.4% vs. 0.0%) (*P* = 0.004). A total of 39 (55.7%) progression-free survival (PFS) events were recorded. The median (95% confidence interval) PFS was 17.0 (14.6-19.4) months after dalpiciclib plus endocrine therapy. The multivariate Cox regression model disclosed that Eastern Cooperative Oncology Group performance status score 2 (vs. 0) [hazard ratio (HR): 4.010, *P* = 0.028], lines of dalpiciclib-based therapy (HR: 4.161, *P* = 0.041), cross-line dalpiciclib (HR: 3.362, *P* = 0.046), and endocrine resistance (HR: 2.728, *P* = 0.047) were associated with shorter PFS. The most common adverse events were leukopenia (97.1%) and neutropenia (95.7%), which were predominantly grade 2 or 3. Grade 4 adverse events included leukopenia (2.9%), neutropenia (7.1%), and thrombopenia (1.4%).

**Conclusion:**

Dalpiciclib plus endocrine therapy possesses acceptable efficacy and safety for the treatment of elderly patients with HR^+^/HER2^-^ advanced breast cancer.

## 1 Introduction

Hormone receptor-positive (HR^+^)/human epidermal growth factor receptor 2-negative (HER2^-^) represents the most frequent molecular subtype of breast cancer, accounting for approximately 70% of all cases (1). Recently, the incidence and prevalence of HR^+^/HER2^-^ advanced breast cancer have grown in elderly individuals, and the mortality is high in this population (1, 2). Regarding treatment, the endocrine regimen remains the mainstay of treatment for HR^+^/HER2^-^ advanced breast cancer, yet its efficacy is often compromised by the development of drug resistance (3–6). On the other hand, elderly patients face several challenges, such as reduced organ function, comorbidities, and lower treatment tolerance, which may impair the efficacy of endocrine therapy and raise safety concerns (7, 8). Therefore, exploring potential adjunctive therapies to endocrine therapy is meaningful for improving the prognosis of elderly patients with HR^+^/HER2^-^ advanced breast cancer.

Dalpiciclib, the first cyclin-dependent kinase 4 and 6 (CDK4/6) inhibitor developed in China, possesses a potent ability to inhibit tumor proliferation (9, 10). A preclinical study reported that dalpiciclib overcame endocrine therapy resistance, and combining dalpiciclib with endocrine therapy exerted remarkable synergistic antitumor activity in HR^+^ breast cancer (11). Furthermore, previous pivotal phase III trials have demonstrated the satisfactory efficacy and safety of dalpiciclib plus endocrine therapy in patients with HR^+^/HER2^-^ breast cancer, reporting median progression-free survival (PFS) ranging from 15.7 to 30.6 months with a low incidence of serious adverse events (12, 13). However, real-world evidence is still inadequate, especially in elderly populations.

Accordingly, this real-world study intended to explore the efficacy and safety of dalpiciclib plus endocrine therapy in elderly patients with HR^+^/HER2^-^ advanced breast cancer.

## 2 Materials and methods

### 2.1 Study subjects

This study retrospectively analyzed data from 70 elderly patients (≥65 years) with HR^+^/HER2^-^ advanced breast cancer who received dalpiciclib plus endocrine therapy. All patients were admitted to the Affiliated Tumor Hospital of Xinjiang Medical University between May 1, 2022, and October 31, 2024. Eligible patients were consecutively included during the study period. The inclusion criteria were as follows (1): age ≥65 years (2); postoperative recurrent or metastatic stage IV breast cancer confirmed by relevant imaging examinations or diagnosed with inoperable stage IV breast cancer (3); estrogen receptor (ER) positive and/or progesterone receptor (PR) positive (14), and HER2 negative or low expression (15) (see **Supplementary Table 1** for interpretation criteria) (4); Eastern Cooperative Oncology Group (ECOG) performance status ≤2 (5); expected survival ≥3 months (6); received at least 2 cycles of dalpiciclib plus endocrine therapy; and (7) adequate organ function. Patients were excluded if they had a history of prior dalpiciclib treatment or incomplete treatment-related information. No additional exclusion criteria were applied. This study received approval from the Ethics Committee of the Affiliated Tumor Hospital of Xinjiang Medical University.

### 2.2 Data collection

Patients’ clinical data, including age, sex, ethnicity, pathological subtype, HR status, HER2 status, molecular subtype, ECOG performance status, tumor stage, visceral metastasis, bone metastasis, metastatic site, number of metastatic sites, salvage chemotherapy, salvage endocrine therapy, lines of dalpiciclib-based therapy, endocrine therapy sensitivity, cross-line dalpiciclib, and combined drugs, were collected. The classification of endocrine therapy sensitivity was based on the advanced breast cancer International Consensus Guidelines (16). Cross-line dalpiciclib was defined as the use of dalpiciclib in patients who had previously received a different CDK4/6 inhibitor.

### 2.3 Treatments

All patients received oral dalpiciclib isethionate tablets (trade name: AiRuiKang, 150 mg/tablet, 125 mg/tablet, or 50 mg/tablet, Jiangsu Hengrui Medicine Co., Ltd.) combined with endocrine therapy. Dalpiciclib was administered orally once daily at a dose of 150 mg for 3 consecutive weeks followed by 1 week off (28-day cycle). Dose reductions to 125 mg or 100 mg once daily were permitted based on patient tolerability. Among the 9 patients with ECOG performance status score 2, 6 patients initiated dalpiciclib treatment with a reduced dose, including 1 patient who started at 125 mg and 5 patients who started at 100 mg. Endocrine therapy regimens were as follows: letrozole (2.5 mg, oral administration, once daily); anastrozole (1 mg, oral administration, once daily); exemestane (25 mg, oral administration, once daily); tamoxifen (10 mg, oral administration, twice daily); and fulvestrant (500 mg, intramuscular injection, once every 28 days, with an additional 500 mg dose administered two weeks after the initial injection).

### 2.4 Evaluation

Efficacy evaluation was performed every 2 cycles, while after 12 months, the interval was changed to every 3 cycles. Patients were instructed to return to the hospital for timely reassessment if disease progression was suspected or confirmed. Treatment responses were assessed via Response Evaluation Criteria in Solid Tumors 1.1 (RECIST 1.1). Additionally, the objective response rate (ORR) and disease control rate (DCR) were calculated. Adverse events were graded using the National Cancer Institute’s Common Terminology Criteria for Adverse Events, version 4.0 (CTCAE v4.0), and managed according to the expert consensus on the management of adverse events of CDK4/6 inhibitors in breast cancer (17).

### 2.5 Follow-up information

Follow-up information was collected via outpatient visits and telephone interviews. PFS was calculated, which was defined as the time from the initiation of dalpiciclib to the confirmation of disease progression or death due to any cause. As of December 31, 2024, 31 patients discontinued dalpiciclib treatment due to disease progression, and their PFS data were reported; 6 patients discontinued the treatment due to drug-related toxicity; 2 patients discontinued the treatment for personal reasons; 31 patients remained on dalpiciclib treatment.

### 2.6 Statistical analysis

Data analysis was performed with SPSS (v25.0) and R (v4.0.3). Patients with missing data were excluded from the analysis. Data were summarized using descriptive statistics, with categorical variables expressed as frequencies and percentages. Comparisons of treatment responses were carried out with the chi-square test. PFS was evaluated through Kaplan-Meier analysis, and the log-rank test was used to compare PFS between patients with different clinical characteristics. To identify factors influencing PFS, both univariate and multivariate Cox proportional hazards regression models were applied. The multivariate analysis was performed to adjust for potential confounding factors. Variables with *P* < 0.05 in the univariate analysis were selected for inclusion in the multivariate model, including ECOG performance status, lines of dalpiciclib-based therapy, cross-line dalpiciclib, endocrine resistance, and newly diagnosed stage IV. A *P* < 0.05 was considered statistically significant.

## 3 Results

### 3.1 Patient selection

A total of 78 patients were initially screened for eligibility between May 1, 2022, and October 31, 2024. Among them, 4 patients were excluded because follow-up data were unavailable, and another 4 patients were excluded because they did not meet the inclusion criteria or met the exclusion criteria. Therefore, 70 patients were ultimately included in the analysis (**Supplementary Figure 1**).

### 3.2 Clinical features and treatment information

A total of 39 (55.7%), 26 (37.1%), and 5 (7.1%) patients were aged ≥65 and <70 years, ≥70 and <80 years, and ≥80 years, respectively. There were 1 (1.4%) male and 69 (98.6%) females. The ECOG performance status score was 0, 1, and 2 in 32 (45.7%), 29 (41.4%), and 9 (12.9%) patients, respectively. Moreover, 24 (34.3%), 27 (38.6%), 16 (22.9%), and 3 (4.3%) elderly patients had Charlson Comorbidity Index (CCI) scores of 2, 3, 4, and ≥5, respectively. The detailed clinical information is shown in **Table 1**. Regarding treatment information, 41 (58.6%) patients received first-line dalpiciclib-based therapy, and 29 (41.4%) patients received second-line or above dalpiciclib-based therapy. Fifty-two (74.3%) patients were sensitive to endocrine therapy, and 18 (25.7%) patients were resistant. Among the resistant patients, 11 (15.7%) had primary resistance and 7 (10.0%) had secondary resistance. Six (8.6%) patients received cross-line dalpiciclib. Among these 6 patients, 2 (2.9%) patients had a history of abemaciclib and 4 (5.7%) patients had a history of palbociclib. The detailed treatment information is shown in **Table 2**.

**Table 1:** Baseline characteristics of the 70 breast cancer patients.

Characteristics	Cases (%)
Age	
≥65 and <70 years	39 (55.7)
≥70 and <80 years	26 (37.1)
≥80 years	5 (7.1)
Sex	
Male	1 (1.4)
Female	69 (98.6)
Ethnicity	
Han	46 (65.7)
Minority	24 (34.3)
Pathological subtype	
Invasive ductal carcinoma	67 (95.7)
Others	3 (4.3)
HR status	
ER positive, PR positive	62 (88.6)
ER positive, PR negative	8 (11.4)
HER2 status	
Negative	25 (35.7)
+	18 (25.7)
++	27 (38.6)
Molecular subtype	
Luminal A	24 (34.3)
Luminal B	46 (65.7)
ECOG performance status	
0	32 (45.7)
1	29 (41.4)
2	9 (12.9)
Newly diagnosed stage IV	
Yes	22 (31.4)
No	48 (68.6)
Visceral metastasis	
Yes	44 (62.9)
No	26 (37.1)
Bone metastasis only	
Yes	16 (22.9)
No	29 (41.4)
Metastatic site	
Liver	16 (22.9)
Lung	34 (48.6)
Brain	2 (2.9)
Kidney	3 (4.3)
Lymph node	17 (24.3)
Pleura	8 (11.4)
Pericardium	1 (1.4)
Contralateral breast	1 (1.4)
Chest wall skin	6 (8.6)
Number of metastatic sites	
1	32 (45.7)
2	18 (25.7)
≥3	20 (28.6)
CCI	
1	0 (0.0)
2	24 (34.3)
3	27 (38.6)
4	16 (22.9)
5	3 (4.3)

HR, hormone receptor; ER, estrogen receptor; PR, progesterone receptor; HER2, human epidermal growth factor receptor 2; ECOG, Eastern Cooperative Oncology Group; CCI, Charlson Comorbidity Index.

**Table 2:** Treatment information.

Items	Cases (%)
Salvage chemotherapy	
No	54 (77.1)
First line	11 (15.7)
Second line or above	5 (7.1)
Salvage endocrine therapy	
No	49 (70.0)
First line	15 (21.4)
Second line or above	6 (8.6)
Lines of dalpiciclib-based therapy	
First line	41 (58.6)
Second line or above	29 (41.4)
Endocrine therapy sensitivity	
Sensitive	52 (74.3)
Resistant	18 (25.7)
Primary resistant	11 (15.7)
Secondary resistant	7 (10.0)
Cross-line dalpiciclib	
Yes	6 (8.6)
No	64 (91.4)
Combined drugs	
Fulvestrant	34 (48.6)
AI	35 (50.0)
letrozole	20 (28.6)
Anastrozole	1 (1.4)
Exemestane	14 (20.0)
Tamoxifen	1 (1.4)
Previous CDK4/6 inhibitors	
Abemaciclib	2 (2.9%)
Palbociclib	4 (5.7%)

AI, aromatase inhibitor; CDK4/6, cyclin-dependent kinase 4 and 6.

The initial doses were 150, 125, and 100 mg/day in 34 (48.6%), 24 (34.3%), and 12 (17.1%) patients, respectively. A total of 7 (10.0%), 5 (7.1%), and 8 (11.4%) patients experienced dose reductions from 150 to 125 mg/day, 150 to 100 mg/day, and 125 to 100 mg/day, respectively. The final maintenance doses were 150, 125, and 100 mg/day in 22 (31.4%), 23 (32.9%), and 25 (35.7%) patients, respectively (**Supplementary Table 2**).

### 3.3 Treatment response

All 70 patients completed the efficacy evaluation. No patients achieved a complete response (CR). A total of 10 (14.3%) and 56 (80.0%) patients achieved a partial response (PR) and stable disease (SD). Four (5.7%) patients experienced progressive disease (PD). The ORR was 14.3%, and the DCR was 94.3% (**Table 3**).

**Table 3:** Tumor response.

Items	Cases (%)
CR	0 (0.0)
PR	10 (14.3)
SD	56 (80.0)
PD	4 (5.7)
ORR	10 (14.3)
DCR	66 (94.3)

CR, complete response; PR, partial response; SD, stable disease; PD, progressive disease; ORR, objective response rate; DCR, disease control rate.

### 3.4 Comparison of treatment response among patients with different clinical features

The ORR was higher in patients with first-line dalpiciclib-based therapy than in those with second-line therapy or above (24.4% vs. 0.0%) (*P* = 0.004). DCR was numerically higher in patients with first-line dalpiciclib-based therapy than in those with second-line therapy or above, but it did not achieve statistical significance (100.0% vs. 90.2%) (*P* = 0.083). The ORR and DCR were not different in patients with other clinical features, including age, salvage chemotherapy, endocrine therapy sensitivity, cross-line dalpiciclib, and final maintenance dose (all *P*>0.05) (**Table 4**).

**Table 4:** Association of clinical features with treatment response.

Characteristics	ORR (%)	*P*-value	DCR (%)	*P*-value
Age		0.491		0.772
≥65 and <70 years	18.0		94.9	
≥70 and <80 years	11.5		92.3	
≥80 years	0.0		100.0	
Lines of dalpiciclib-based therapy		0.004		0.083
First line	24.4		90.2	
Second line or above	0.0		100.0	
Salvage chemotherapy		0.063		0.262
Yes	0.0		100.0	
No	18.5		92.6	
Endocrine therapy sensitivity		0.656		0.252
Sensitive	15.4		96.2	
Resistant	11.1		88.9	
Cross-line dalpiciclib		0.296		0.528
Yes	0.0		100.0	
No	15.6		93.8	
Final maintenance dose		0.373		0.376
150 mg	22.7		100.0	
125 mg	8.7		91.3	
100 mg	12.0		88.0	

ORR, objective response rate; DCR, disease control rate.

### 3.5 Survival

**Figure 1:**
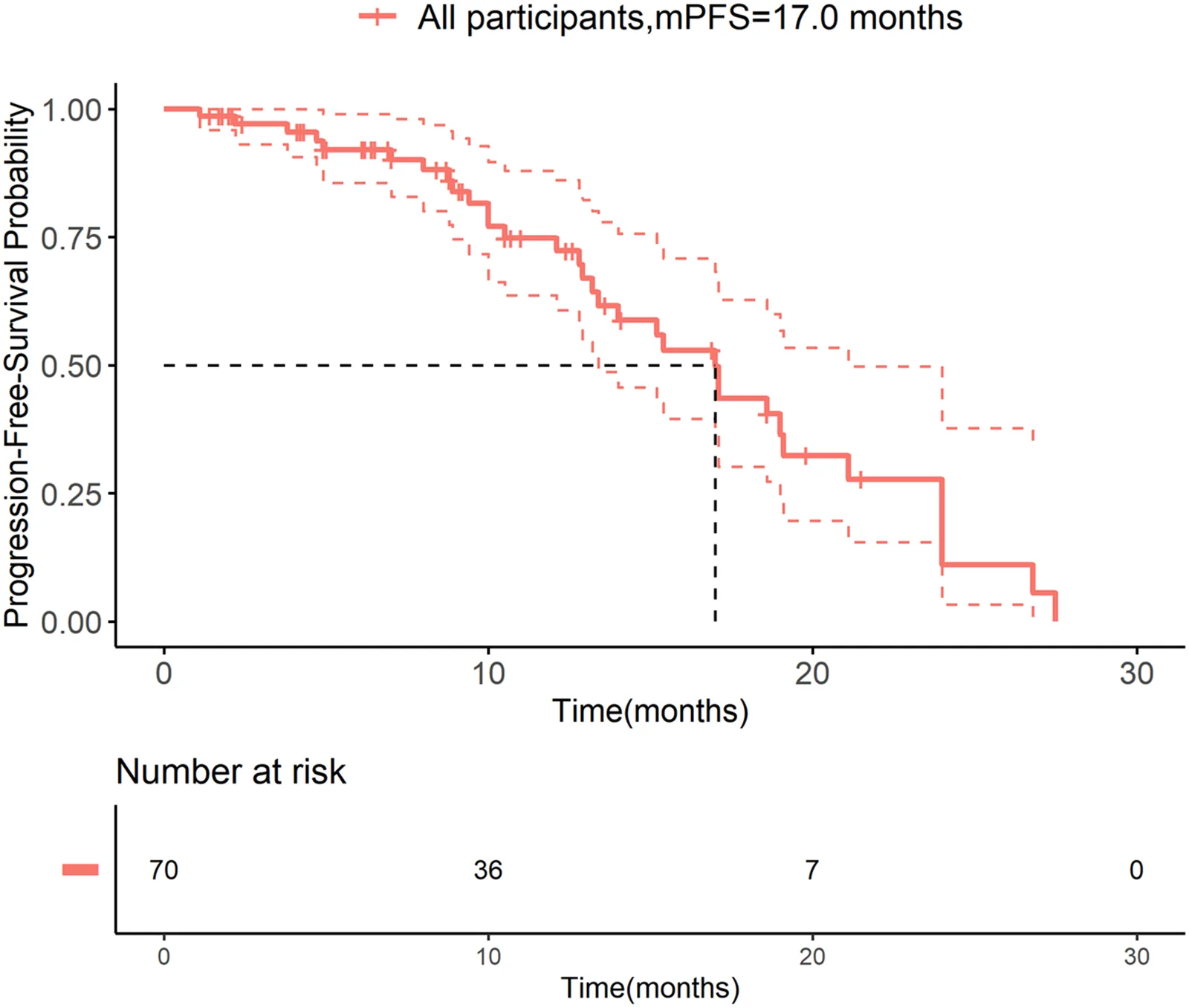
The median PFS in all patients. A total of 37 patients were censored.

The median (range) follow-up duration was 13.5 (1.7-29.4) months. A total of 39 (55.7%) patients experienced disease progression or death. The median (95% CI) PFS was 17.0 (14.6-19.4) months (**Figure 1**). Five (7.1%) patients died. The median overall survival (OS) was not reached.

### 3.6 Comparison of survival among patients with different clinical features

**Figure 2:**
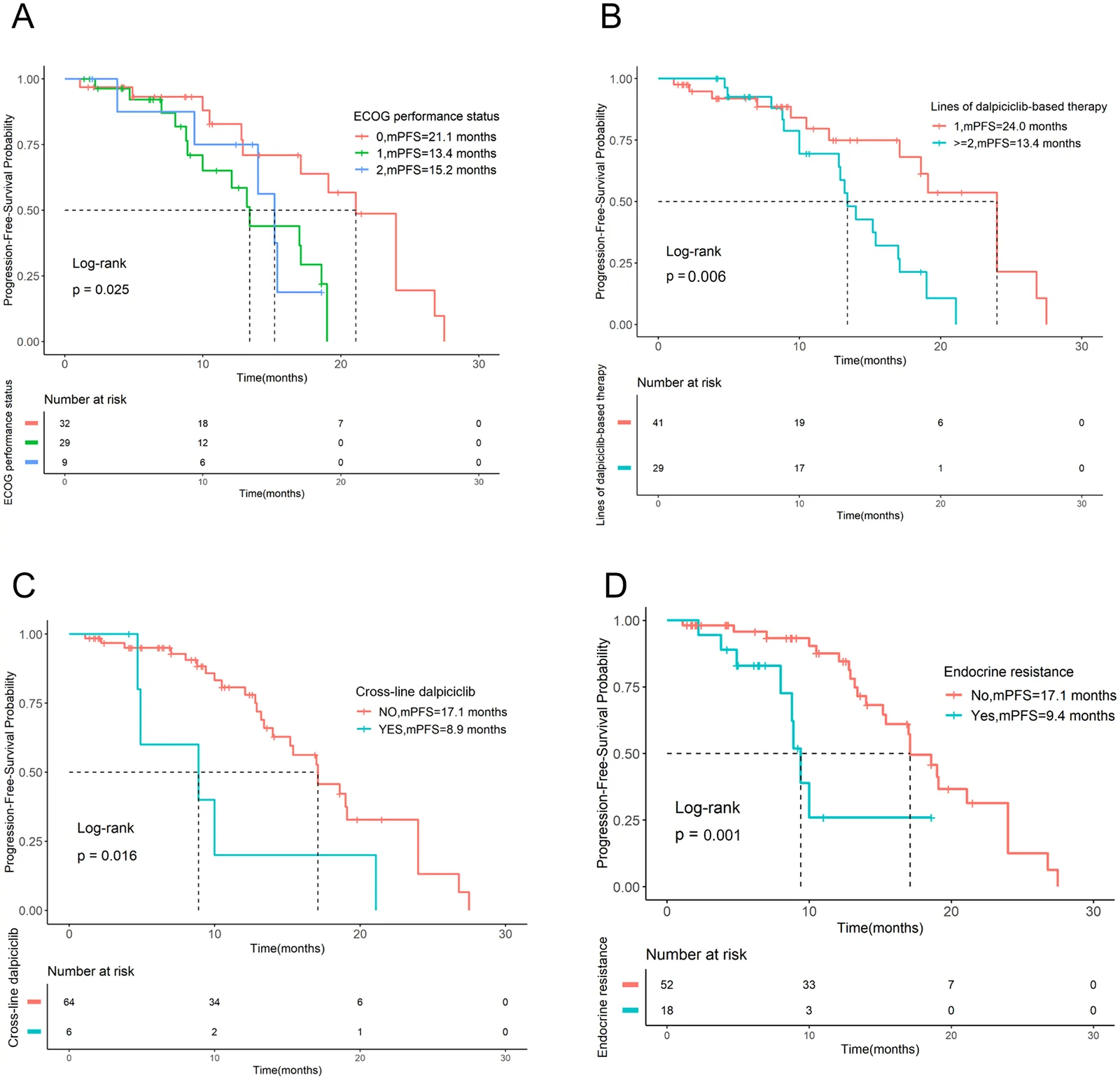
Subgroup analyses for PFS. Association of ECOG performance status **(A)**, lines of dalpiciclib-based therapy **(B)**, cross-line dalpiciclib **(C)**, and endocrine resistance **(D)** with PFS. The numbers of censored patients were 28, 15, and 4 for ECOG performance status 0, 1, and 2, respectively; 26 and 11 for first-line therapy and second-line therapy or above, respectively; 36 and 1 for patients without and with cross-line dalpiciclib, respectively; 27 and 10 for patients without and with endocrine resistance, respectively.

PFS was prolonged in patients with an ECOG performance status score of 0, followed by patients with ECOG performance status scores of 2 and 1 (*P* = 0.025). The median PFS was 21.1, 13.4, and 15.2 months in patients with ECOG performance status scores of 0, 1, and 2, respectively (**Figure 2A**).

PFS was prolonged in patients receiving first-line dalpiciclib-based therapy vs. those receiving second-line therapy or above (*P* = 0.006). The median PFS was 24.0 and 13.4 months in patients receiving first-line and second-line or above dalpiciclib-based therapy, respectively (**Figure 2B**).

PFS was prolonged in patients who did not receive cross-line dalpiciclib compared to those who received cross-line dalpiciclib (*P* = 0.016). The median PFS was 17.1 months in patients who did not receive cross-line dalpiciclib and 8.9 months in patients who received cross-line dalpiciclib. It should be clarified that only 6 patients received cross-line dalpiciclib; therefore, these findings should be interpreted with caution due to the very limited sample size and should be considered exploratory in nature (**Figure 2C**).

PFS was longer in patients without endocrine resistance than in those with endocrine resistance (*P* = 0.001). The median PFS was 17.1 and 9.4 months in patients without and with endocrine resistance, respectively (**Figure 2D**).

### 3.7 Factors related to survival

Univariate Cox regression analysis suggested that ECOG performance status score 1 (vs. 0) (*P* = 0.011), lines of dalpiciclib-based therapy (*P* = 0.008), cross-line dalpiciclib (*P* = 0.023), endocrine resistance (*P* = 0.002), and newly diagnosed stage IV (*P* = 0.045) were related to poor PFS.

In the multivariate Cox regression analysis, ECOG performance status score 2 (vs. 0) [hazard ratio (HR): 4.010, *P* = 0.028], lines of dalpiciclib-based therapy (HR: 4.161, *P* = 0.041), cross-line dalpiciclib (HR: 3.362, *P* = 0.046), and endocrine resistance (HR: 2.728, *P* = 0.047) were associated with shorter PFS (**Table 5**).

**Table 5:** Univariate and multivariate Cox regression analysis for PFS.

Characteristics	Univariate Cox regression			Multivariate Cox regression		
	HR	95% CI	*P*-value	HR	95% CI	*P*-value
Age	0.650	0.301-1.403	0.272			
Ethnicity	1.412	0.635-3.137	0.397			
Pathological subtype	1.696	0.397-7.249	0.476			
Family history	2.045	0.468-8.942	0.342			
ECOG performance status						
0 (reference)	1.000	(-)	(-)	1.000	(-)	(-)
1	3.326	1.316-8.407	0.011	2.648	0.952-7.368	0.062
2	2.841	0.874-9.233	0.083	4.010	1.165-13.799	0.028
PR	1.045	0.311-3.505	0.944			
HER2 status						
Negative (reference)	1.000	(-)	(-)	1.000	(-)	(-)
+	1.585	0.669-3.752	0.295			
++	1.031	0.441-2.410	0.944			
Molecular subtype	1.511	0.709-3.223	0.285			
Number of metastatic sites						
1 (reference)	1.000	(-)	(-)	1.000	(-)	(-)
2	0.656	0.282-1.523	0.326			
≥3	1.230	0.519-2.915	0.638			
Liver metastasis	1.491	0.665-3.341	0.332			
Lung metastasis	1.070	0.531-2.155	0.850			
Pleural metastasis	1.133	0.338-3.792	0.840			
Bone metastasis	1.022	0.455-2.296	0.958			
Lymph node metastasis	0.509	0.177-1.461	0.210			
Skin metastasis	1.006	0.303-3.344	0.992			
Kidney metastasis	0.788	0.106-5.841	0.815			
Visceral metastasis	1.076	0.519-2.231	0.843			
Lines of dalpiciclib-based therapy	2.927	1.318-6.498	0.008	4.161	1.061-16.317	0.041
Combined drugs						
Fulvestrant (reference)	1.000	(-)	(-)	1.000	(-)	(-)
Letrozole	0.833	0.337-2.063	0.694			
Others	1.307	0.557-3.068	0.538			
Cross-line dalpiciclib	3.119	1.172-8.301	0.023	3.362	1.020-11.083	0.046
Endocrine resistance	4.109	1.661-10.164	0.002	2.728	1.015-7.355	0.047
Whether first medication	1.870	0.874-4.002	0.100			
Initial dose						
100 mg (reference)	1.000	(-)	(-)	1.000	(-)	(-)
125 mg	2.477	0.831-7.379	0.103			
150 mg	1.967	0.697-5.549	0.201			
Final maintenance dose						
100 mg (reference)	1.000	(-)	(-)	1.000	(-)	(-)
125 mg	1.453	0.625-3.380	0.385			
150 mg	1.710	0.703-4.159	0.236			
Newly diagnosed stage IV	2.706	1.022-7.163	0.045	2.213	0.804-6.090	0.124

PFS, progression-free survival; HR, hazard ratio; CI, confidence interval; ECOG, Eastern Cooperative Oncology Group; PR, progesterone receptor; HER2, human epidermal growth factor receptor 2.

### 3.8 Safety

The most common adverse events after dalpiciclib plus endocrine therapy were hematological toxicities, primarily leukopenia (97.1%) and neutropenia (95.7%); most of them were grades 2-3. Grade 4 hematological toxicities included leukopenia, neutropenia, and thrombopenia, occurring in 2.9%, 7.1%, and 1.4% of patients, respectively. Regarding non-hematological toxicities, increased blood creatinine was the most common adverse event (12.9%), all of which were grade 1. Fatigue (8.6%) was the second most common non-hematological toxicity, predominantly grade 3. No grade 4 non-hematological toxicities occurred (**Table 6**).

**Table 6:** Adverse events.

Adverse reactions	Cases (%)	Grade (%)			
		1	2	3	4
Hematological toxicities						
Leukopenia	68 (97.1)	12 (17.1)	30 (42.9)	24 (34.3)	2 (2.9)
Neutropenia	67 (95.7)	6 (8.6)	27 (38.6)	29 (41.4)	5 (7.1)
Anaemia	25 (35.7)	14 (20.0)	9 (12.9)	2 (2.9)	0 (0.0)
Thrombopenia	19 (27.1)	12 (17.1)	3 (4.3)	3 (4.3)	1 (1.4)
Non-hematological toxicities						
Nausea	3 (4.3)	3 (4.3)	0 (0.0)	0 (0.0)	0 (0.0)
Diarrhea	2 (2.9)	2 (2.9)	0 (0.0)	0 (0.0)	0 (0.0)
Fatigue	6 (8.6)	2 (2.9)	1 (1.4)	3 (4.3)	0 (0.0)
Stomatitis	1 (1.4)	1 (1.4)	0 (0.0)	0 (0.0)	0 (0.0)
Rash	2 (2.9)	1 (1.4)	1 (1.4)	0 (0.0)	0 (0.0)
Increased AST/ALT	4 (5.7)	2 (2.9)	1 (1.4)	1 (1.4)	0 (0.0)
Increased blood creatinine	9 (12.9)	9 (12.9)	0 (0.0)	0 (0.0)	0 (0.0)

AST, aspartate aminotransferase; ALT, alanine aminotransferase.

No patients experienced treatment interruption in this study. Treatment delay occurred in 32 (45.7%) patients, granulocyte colony-stimulating factor (G-CSF) was used in 14 (20.0%) patients, and 3 (4.3%) patients were hospitalized due to treatment-related toxicity. The main causes of treatment delay included neutropenia (29 cases) and thrombocytopenia (3 cases), and the median delay duration was 7 days (range: 3–14 days). G-CSF was used for therapeutic purposes, and the indications included grade 4 neutropenia (5 cases) and grade 3 neutropenia (9 cases). Overall, these findings suggested that treatment delays and G-CSF use were mainly attributable to manageable hematologic toxicities and were generally controllable in this elderly population. The details of these safety outcomes are shown in **Supplementary Table 3**.

## 4 Discussion

Combination therapy involving endocrine agents and CDK4/6 inhibitors represents the cornerstone of treatment for patients with HR^+^/HER2^-^ advanced breast cancer (18, 19). As a novel CDK4/6 inhibitor, dalpiciclib also exhibits satisfactory efficacy (12, 13, 20, 21). Previous phase III trials and real-world clinical studies demonstrated that dalpiciclib plus endocrine therapy achieved an ORR of 26.9%-57.4% and a DCR of 86.1%-88.7% in patients with HR^+^/HER2^-^ advanced breast cancer (12, 13, 20, 21). In our study, we found that dalpiciclib plus endocrine therapy achieved an ORR and DCR of 14.3% and 94.3%, respectively, in elderly patients. The ORR in this study was lower than that of previous studies, but the DCR was higher (12, 13, 20, 21). The discrepancies in treatment response may be related to diverse baseline characteristics of patients, such as lines of treatment and age. Overall, our study provides insights that dalpiciclib plus endocrine therapy could effectively control tumor progression in elderly patients (as reflected by the high DCR of 94.3%). Moreover, we found that ORR was higher in patients receiving first-line dalpiciclib plus endocrine therapy than in those receiving second-line therapy or above. A similar trend was found for DCR, but it did not achieve statistical significance. These findings suggest that the use of dalpiciclib plus endocrine therapy in the first-line setting may be associated with improved outcomes in elderly patients with HR^+^/HER2^-^ advanced breast cancer.

The DAWNA-1 and DAWNA-2 studies reported that the median PFS after dalpiciclib plus endocrine therapy ranged from 15.7 to 30.6 months in patients with HR^+^/HER2^-^ advanced breast cancer (12, 13). A real-world study found that dalpiciclib plus endocrine therapy achieved a median PFS of 18.0 months in patients with HR^+^/HER2^-^ advanced breast cancer (20). In the current study, the median PFS was 17.0 months after treatment with dalpiciclib plus endocrine therapy in elderly patients, which was consistent with the PFS in broader patient populations in previous studies (12, 13, 20). However, such comparisons should be interpreted with caution due to differences in patient age distribution, treatment lines, and the inherent heterogeneity between clinical trial and real-world populations. Regarding other CDK4/6 inhibitors, previous studies have found that these regimens could achieve favorable PFS in broad patient populations (20, 22–26). In elderly patients, previous real-world studies reported that CDK4/6 inhibitors (including palbociclib, ribociclib, and abemaciclib) plus endocrine therapy achieved a median PFS of 13.4 to 28.0 months (27–29). In comparison, the median PFS of 17.0 months observed in our study fell within the previously reported data, suggesting the comparable efficacy of dalpiciclib with other CDK4/6 inhibitors in elderly patients.

To identify patients who may be at risk of poor prognosis after dalpiciclib plus endocrine therapy, this study further explored factors related to PFS (1). ECOG performance status score 2 was related to poor PFS. This finding indicated that elderly patients with poor physical conditions might benefit less from dalpiciclib plus endocrine therapy. Of note, the Kaplan-Meier curves showed that the ECOG 2 subgroup appeared to have a longer PFS than the ECOG 1 subgroup. This unexpected observation might be due to the limited sample size and potential imbalance in baseline characteristics between ECOG subgroups (2). Elevated lines of dalpiciclib-based therapy were associated with poor PFS. This finding was in line with previous studies (30–32) (3). Cross-line dalpiciclib was associated with shorter PFS. Our finding was inconsistent with the previous phase III postMONARCH trial (33), which demonstrated that switching to abemaciclib plus fulvestrant after progression on prior CDK4/6 inhibitor-based therapy still improved PFS compared with endocrine therapy alone in patients with advanced breast cancer. We speculated that this discrepancy might be primarily attributed to the very limited number of patients receiving cross-line dalpiciclib in our study (n=6), which might affect the statistical power. Therefore, our finding should be interpreted with caution and considered exploratory. Further studies with larger sample sizes are needed to clarify the potential impact of treatment sequencing on clinical outcomes (4). Endocrine resistance was related to unsatisfactory PFS. This finding was in line with a previous study (20). A potential reason might be that endocrine resistance might impair the antitumor effect of endocrine agents in the dalpiciclib-based combination regimen, thereby worsening PFS (34).

Dalpiciclib possesses an acceptable safety profile in patients with HR^+^/HER2^-^ advanced breast cancer (12, 13, 20). Previous studies reported that the most frequent adverse events after dalpiciclib plus endocrine therapy were hematological toxicities, primarily neutropenia and leukopenia (12, 13, 20). Consistent with the findings of previous studies, we also discovered that neutropenia and leukopenia were the most frequent adverse events after dalpiciclib plus endocrine therapy in elderly patients. The majority of neutropenia and leukopenia were grade 2-3, and the incidence of grade 4 neutropenia (7.1%) and leukopenia (2.9%) was low. In addition, we found that the incidence of non-hematological toxicities was relatively low, with increased blood creatinine and fatigue being the most common adverse events after dalpiciclib plus endocrine therapy. No grade 4 non-hematological toxicities were discovered. Overall, the adverse events of dalpiciclib in elderly patients were manageable, suggesting the acceptable safety profile of this CDK4/6 inhibitor. In this study, 20 patients had dose reduction due to adverse events, of which 5 patients required consecutive dose reductions. As reported by a previous study, dose modifications of CDK4/6 inhibitors in elderly patients were common, but did not appear to compromise PFS (35). Therefore, close monitoring of adverse events and timely dose modification are essential for this frail patient population, and such dose adjustments may help maintain treatment continuity without negatively affecting efficacy.

The combination of CDK4/6 inhibitor and endocrine therapy remains the standard first-line treatment for HR^+^/HER2^-^ advanced breast (36). However, resistance inevitably develops, necessitating alternative therapeutic strategies (36). Endocrine therapies, such as oral selective estrogen receptor degraders (SERDs), show promising efficacy, especially in patients with estrogen receptor 1 (ESR1) mutations (37). Targeted therapies, such as phosphoinositide 3-kinase (PI3K)/protein kinase B (AKT)/mammalian target of rapamycin (mTOR) inhibitors, demonstrate clinical benefit by addressing downstream signaling pathways involved in resistance (37). Furthermore, several novel strategies are under investigation, such as antibody-drug conjugates and targeting CDK7 (37). The above treatment strategies may provide additional therapeutic options for elderly patients with HR^+^/HER2^-^ advanced breast cancer who experience disease progression or develop resistance following dalpiciclib-based therapy.

Several limitations should be noted in this study (1). Due to the retrospective single-center design, selection bias might exist. As all patients were treated at a specialized cancer center, the cohort might not fully represent the broader elderly population with HR^+^/HER2^-^ advanced breast cancer, thereby limiting the generalizability of the findings (2). Only 70 elderly patients were enrolled, indicating a relatively small sample size and limited statistical power; therefore, larger-scale studies are required to further validate the efficacy and safety of dalpiciclib plus endocrine therapy in elderly patients (3). This study lacked a control group, which limited the ability to perform direct comparative analyses and might weaken the strength of the conclusions (4). Due to the small number of patients receiving cross-line dalpiciclib, the observed association between cross-line dalpiciclib and PFS might not be statistically reliable. Therefore, this finding should be further validated in larger, well-designed prospective studies (5). The sample size of this study was relatively small, which might have led to incomplete capture of some adverse events associated with dalpiciclib treatment. Future studies could consider utilizing external databases, such as FAERS, to further complement the analysis of adverse events (6). As this was a retrospective study, comprehensive geriatric assessment parameters, such as activities of daily living, instrumental activities of daily living, nutritional status, and frailty index, were not systematically collected, which might limit a more comprehensive evaluation of outcomes in elderly patients (7). CCI was not included in the multivariable analysis because it could not effectively discriminate between different risk strata in this cohort, with scores largely concentrated between 2 and 4. Including such a variable in a relatively small sample might increase the risk of model overfitting. Therefore, larger-scale studies are needed to further evaluate the association between comorbidity burden and prognosis in elderly patients (8). Information on the duration of prior CDK4/6 inhibitor therapy was not available in this study, which might limit the interpretation of cross-line treatment outcomes (9). Despite multivariable adjustment, the relatively small sample size and limited number of outcome events might increase the risk of model overfitting, and therefore the observed associations should be interpreted with caution (10). Four patients with unavailable follow-up data discontinued treatment because of adverse reactions such as fatigue and poor appetite and were subsequently lost to follow-up. As these patients were excluded from the final analysis, this complete-case approach might introduce selection bias and could potentially lead to an overestimation of treatment efficacy and tolerability (11). Molecular alteration data, including phosphatidylinositol-4,5-bisphosphate 3-kinase catalytic subunit alpha (PIK3CA), ESR1, breast cancer susceptibility genes 1 and 2 (BRCA1/2), and AKT signaling pathway mutations, were not available in this study. These genomic factors might influence the prognosis in elderly patients with HR^+^/HER2^-^ advanced breast cancer and thus represent the potential confounders, particularly in the relationship between endocrine resistance and PFS (12). Post-progression treatment strategies might influence clinical outcomes in elderly patients, but these data were not available in this study. Further studies should consider collecting such data and investigating the impact of post-progression treatment strategies on the prognosis of elderly patients with HR^+^/HER2^-^ advanced breast cancer.

## 5 Conclusion

In conclusion, dalpiciclib plus endocrine therapy exhibits acceptable efficacy and safety in elderly patients with HR^+^/HER2^-^ advanced breast cancer. Clinically, this combined regimen may be considered as a treatment option. However, given the observational nature of this study, further randomized controlled trials are warranted to confirm these findings.

## Data Availability

The original contributions presented in the study are included in the article/**Supplementary Material**. Further inquiries can be directed to the corresponding author.

## Supplementary material

Supplementary Figure 1 Study flow.
