# Supplementary material for: Efficacy and safety of dalpiciclib combined with endocrine therapy in elderly patients with HR+/HER2- advanced breast cancer: a real-world study
Source: Front Oncol. 2026 Aug 18;16:1789316. doi: 10.3389/fonc.2026.1789316 (PMC13529596; doi:10.3389/fonc.2026.1789316)
Supplement: Supplementary file 1 [file Table1.docx]

**Supplementary Table 1.** The interpretation of HER2 testing.

|  | Interpretation of HER2 IHC results | HER2 status |
| --- | --- | --- |
| 0 | No staining or ≤10% of invasive cancer cells show incomplete and weak cell membrane staining. | HER2 negative |
| + | >10% of invasive cancer cells show incomplete and weak cell membrane staining. | HER2 low expression |
| ++ | >10% of invasive cancer cells show weak to moderate intensity, complete cell membrane staining, or ≤10% of invasive cancer cells show strong and complete cell membrane staining. | HER2 IHC result is uncertain, and the HER2 status should be further tested by FISH method: IHC2^+^/FISH^+^ is HER2 positive; IHC2^+^/FISH^-^ is HER2 low expression |
| +++ | >10% of invasive cancer cells show strong, complete, and uniform cell membrane staining. | HER2 positive |

HER2, human epidermal growth factor receptor 2; IHC, immunohistochemistry; FISH, fluorescence in situ hybridization.
