# Supplementary material for: Efficacy and safety of dalpiciclib combined with endocrine therapy in elderly patients with HR+/HER2- advanced breast cancer: a real-world study
Source: Front Oncol. 2026 Aug 18;16:1789316. doi: 10.3389/fonc.2026.1789316 (PMC13529596; doi:10.3389/fonc.2026.1789316)
Supplement: Supplementary file 2 [file Table2.docx]

**Supplementary Table 2.** Dose adjustment of dalpiciclib.

| Dose of dalpiciclib | Cases (%) |
| --- | --- |
| Initial dose (mg/day) |  |
| 150 | 34 (48.6) |
| 125 | 24 (34.3) |
| 100 | 12 (17.1) |
| Dose reduction (mg/day) |  |
| 150 to 125 | 7 (10.0) |
| 150 to 100 | 5 (7.1) |
| 125 to 100 | 8 (11.4) |
| Final maintenance dose (mg/day) |  |
| 150 | 22 (31.4) |
| 125 | 23 (32.9) |
| 100 | 25 (35.7) |
