# Supplementary material for: Efficacy and safety of dalpiciclib combined with endocrine therapy in elderly patients with HR+/HER2- advanced breast cancer: a real-world study
Source: Front Oncol. 2026 Aug 18;16:1789316. doi: 10.3389/fonc.2026.1789316 (PMC13529596; doi:10.3389/fonc.2026.1789316)
Supplement: Supplementary file 3 [file Table3.docx]

**Supplementary Table 3.** Additional safety outcomes.

| Safety outcomes | Cases (%) | Details |
| --- | --- | --- |
| Treatment interruption | 0 (0.0) | No patients discontinued treatment due to adverse events; all patients were able to tolerate therapy after dose reduction. |
| Treatment delay | 32 (45.7) | Main causes included neutropenia (29 cases) and thrombocytopenia (3 cases). The median delay duration was 7 days (range: 3-14 days). |
| G-CSF use | 14 (20.0) | All were therapeutic use. Indications included grade 4 neutropenia (5 cases) and grade 3 neutropenia (9 cases). |
| Hospitalization due to toxicity | 3 (4.3) | Causes included: 1 case of grade 4 thrombocytopenia; 1 case of grade 3 AST/ALT elevation with fatigue; and 1 case of grade 4 neutropenia accompanied by grade 3 thrombocytopenia and grade 3 anemia. |

G-CSF, granulocyte colony-stimulating factor; AST, aspartate aminotransferase; ALT, alanine aminotransferase.
